# Editorial: Molecular targets for anticancer drug discovery and development

**DOI:** 10.3389/fgene.2024.1374867

**Published:** 2024-04-03

**Authors:** Monde Ntwasa, Zodwa Dlamini

**Affiliations:** ^1^ Department of Life and Consumer Sciences, University of South Africa, Florida, South Africa; ^2^ SAMRC Precision Oncology Research Unit (PORU), DSI/NRF SARChI Chair in Precision Oncology and Cancer Prevention (POCP), University of Pretoria, Pretoria, South Africa

**Keywords:** small molecule anticancer drugs, molecularly targeted therapies, pharmacogenetics, pharmacogenomics, model organisms (*Drosophila*), drug repurposing

## Introduction

The advantages of molecularly targeted therapies are apparent and are the force behind the fast-growing area of small molecule anticancer drug discovery and development. There is a growing list of approved targeted therapies with remarkable success rates (Issa et al., 2021; Sun et al., 2021). These treatments can be administered orally and reduce the threat of immune responses, which is a considerable advantage compared to injections. However, there are challenges as orally administered drugs must contend with immense pharmacokinetic barriers such as pre-systemic metabolism. Nevertheless. anticancer small molecule drug discovery and development is an urgent research matter due to the harsh consequences of chemotherapy and radiotherapy. Furthermore, adverse events due to genetic variations in patients demand that pharmacogenetic and pharmacogenomic approaches be adopted in designing new drugs.

Pharmacogenetics and pharmacogenomics have become crucial research areas with advances in personalized medicine and resistance developed during cancer treatments. Genetic variations limit the effectiveness of many interventions where hormonal, molecular targeted, and chemotherapeutic therapies have been applied. Genetic variations within populations sometimes impact the pharmacokinetics and pharmacodynamics of anticancer drugs (Ulrich et al., 2003; Marsh and McLeod, 2004; Tan et al., 2008). In turn, these genetic backgrounds affect the safety and efficacy of anticancer drugs, reinforcing the critical importance of rigorous research on genetic variations in developing personalized medicine (Carr et al., 2021). The variations may be caused by specific genetic lesions or associated with dysregulation of molecular pathways (Ulrich et al., 2003).

Using model organisms is crucial in discovering anticancer drugs and determining safety and efficacy. The application of the model organisms, such as *Drosophila*, is beneficial as the fly can be used in large numbers, is tractable, and provides data from a whole organism compared to cell culture.

Due to the lengthy periods taken to develop anticancer drugs *de novo*, repositioning approved drugs is preferred. This is possible due to the off-target effects that most small-molecule drugs have. The primary advantage of repurposing is the significantly shortened development time. Such repurposed drugs can be combined with other small molecules when there is synergy or complementarity with chemotherapy or radiotherapy. Consequently, the ReDO project, which offers an online database of drugs, is an essential platform for oncology drug developers (Pantziarka et al., 2018). This database was developed by curating licensed drugs with published anticancer activity. The submissions in this collection could contribute to the ReDo project.

## Highlights from contributing literature

A standout in this Research Topic is a research article by Alam et al., presenting a comprehensive bioinformatics study identifying critical molecules associated with breast cancer (BC). Utilizing 3-D structures, the authors screened the PubChem database, identifying 16 repurposable drugs, including mastinib, a tyrosine kinase inhibitor approved for progressive muscle sclerosis. The study pinpointed eight robust BC-associated key genes (*EGFR*, *FN1*, *EZH2*, *MET*, *CDK1*, *AURKA*, *TOP2A*, and *BIRC5*) through bioinformatics and machine learning, delving into their mechanisms, regulatory influences, and prognostic significance. They suggested 16 promising repurposing drug candidates through molecular docking analysis, supported by an extensive literature review. Focusing on masitinib, they unveiled its anti-BC effects by impeding the mTOR signaling pathway and inducing apoptotic cell death. The findings suggest potential diagnostic and therapeutic strategies for BC.

A comprehensive study by Lee et al., reveals critical insights into genetic variants associated with gastric cancer (GC) and identifies potential drug target candidates relevant to anti-GC drug development. Following their exhaustive investigation and multifaceted functional analysis of reported genetic variants associated with GC, their findings strongly suggested that PRKAA1 emerged as a pivotal gene in the development of GC. Their results indicated that PRKAA1, integral to the PI3K-Alt-mTOR signaling pathway, holds promise as a potential target for future drug development to address GC, share insights into deploying machine learning in elucidating target molecules and pathways that may be amenable to drug design.

Elebo et al. present insights into the predictive value of molecular subtypes in treating pancreatic ductal adenocarcinoma (PDAC). Moreover, the article covers repurposed small-molecule drugs in preclinical and clinical studies. PDAC, whose survival rate is less than 5 years, remains one of the most critical cancers for urgent clinical attention, especially when it comes to diagnostic techniques. The authors conclude that the stratification of PDAC into clinically and genetically linked groups can potentially unveil novel biomarkers. They further state that embracing advanced technologies such as single-cell RNA sequencing and single-cellomics could offer a more exhaustive classification of PDAC patients, delineating subtypes based on their biological characteristics, prognosis, therapeutic targets, and pharmacological drug responses.

Munnik et al., show that *Drosophila melanogaster* is a crucial platform in anticancer drug discovery and development, primarily as a screening platform and validating the drugs at the preclinical stage. The power of *Drosophila* is the genetic tractability in modeling a diverse range of cancers. The authors briefly explain that *Drosophila* possesses conserved genes, low redundancy, easy genetic manipulation, and a rapid life cycle. They suggest that *Drosophila*’s cost-effectiveness and ability to generate cancer models tailored to specific genetic requirements make them suitable for high-throughput screening of anticancer therapies. They further draw our attention to the fact that *Drosophila* models can assess FDA-approved drugs, study bioavailability and toxicity, and facilitate personalized therapy for cancers with multiple genetic mutations. In conclusion, the authors confirm that despite being in its early stages, heightened awareness and increased utilization of *Drosophila* in drug discovery can markedly decrease both the time and cost associated with developing anticancer drugs.

## Conclusion

**FIGURE 1:**
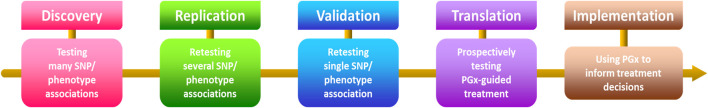
Steps involved in the process of discovering and implementing pharmacogenetics and pharmacogenomics. SNP - single nucleotide polymorphism, PGx—pharmacogenetics and pharmacogenomics.

In conclusion, this comprehensive collection fervently dedicates itself to identifying potential drug targets and advancing anticancer drug development. The accepted articles significantly contribute to the expansive field, focusing primarily on small-molecule drug discovery, with an emphasis on Pharmacogenetics and Pharmacogenomics in anticancer-targeted research. Noteworthy advancements showcased in the contributions promise to catalyze further progress, guiding both researchers and practitioners. By intricately exploring pharmacogenetic and pharmacogenomic factors (Figure 1), the collection illuminates potential drug targets, laying a foundation for tailored and efficacious anticancer therapies. Ultimately, this collection aspires to shape the future of anticancer therapeutics, offering valuable insights and propelling innovation within the scientific community.

## References

[B1] Carr D. F. Turner R. M. Pirmohamed M. (2021). Pharmacogenomics of anticancer drugs: personalising the choice and dose to manage drug response. Br. J. Clin. Pharmacol. 87, 237–255. 10.1111/bcp.14407 32501544

[B2] Issa N. T. Stathias V. Schürer S. Dakshanamurthy S. (2021). Machine and deep learning approaches for cancer drug repurposing. Semin. Cancer Biol. 68, 132–142. 10.1016/j.semcancer.2019.12.011 31904426 PMC7723306

[B3] Marsh S. McLeod H. L. (2004). Cancer pharmacogenetics. Br. J. Cancer 90, 8–11. 10.1038/sj.bjc.6601487 14710198 PMC2395337

[B4] Pantziarka P. Verbaanderd C. Sukhatme V. Rica Capistrano I. Crispino S. Gyawali B. (2018). ReDO_DB: the repurposing drugs in oncology database. Ecancermedicalscience 12, 886. 10.3332/ecancer.2018.886 30679953 PMC6345075

[B5] Sun G. Rong D. Li Z. Sun G. Wu F. Li X. (2021). Role of small molecule targeted compounds in cancer: progress, opportunities, and challenges. Front. Cell Dev. Biol. 9. 10.3389/fcell.2021.694363 PMC8455877 34568317

[B6] Tan S.-H. Lee S.-C. Goh B.-C. Wong J. (2008). Pharmacogenetics in breast cancer therapy. Clin. Cancer Res. 14, 8027–8041. 10.1158/1078-0432.CCR-08-0993 19088019

[B7] Tridente G. Jana A. Nath A. Ashraf G. M. (2023). “Protein kinase inhibitors as therapeutics in neurodegenerative and psychiatric disorders,” in Receptor tyrosine kinases in neurodegenerative and psychiatric disorders (Amsterdam, Netherlands: Elsevier), 403–573. 10.1016/B978-0-443-18677-6.00015-4

[B8] Ulrich C. M. Robien K. McLeod H. L. (2003). Cancer pharmacogenetics: polymorphisms, pathways and beyond. Nat. Rev. Cancer 3, 912–920. 10.1038/nrc1233 14740638

[B9] Vermersch P. Brieva-Ruiz L. Fox R. J. Paul F. Ramio-Torrenta L. Schwab M. (2022). Efficacy and safety of masitinib in progressive forms of multiple sclerosis. Neurol. Neuroimmunol. Neuroinflamm 9. 10.1212/NXI.0000000000001148 PMC9005047 35190477

